# Knowing in general dental practice: Anticipation, constraint, and collective bricolage

**DOI:** 10.1111/jep.13051

**Published:** 2018-10-18

**Authors:** Dominic Hurst, Trish Greenhalgh

**Affiliations:** ^1^ Queen Mary University of London, Barts and The London School of Medicine and Dentistry, Dental Hospital Turner Street London E1 2AD UK; ^2^ Nuffield Department of Primary Care Health Sciences University of Oxford Radcliffe Primary Care Building, Radcliffe Observatory Quarter, Woodstock Road Oxford OX2 6GG UK

**Keywords:** general dental practice, knowledge, practice epistemology, practice theory, sociomateriality, video

## Abstract

**Rationale, aims, and objectives:**

Much of the literature concerned with health care practice tends to focus on a decision‐making model in which knowledge sits within the minds and bodies of health care workers. Practice theories de‐centre knowledge from human actors, instead situating *knowing* in the interactions between all human and non‐human actors. The purpose of this study was to explore how practice arises in the moment‐to‐moment interactions between general dental practitioners (GDPs), patients, nurses, and things.

**Method:**

Eight GDPs in two dental practices, their respective nurses, 23 patients, and material things were video‐recorded as they interacted within clinical encounters. Videos were analysed using a performative approach. Several analytic methods were used: coding of interactions in‐video; pencil drawings with transcripts; and dynamic transcription. These were used pragmatically and in combination. Detailed reflective notes were recorded at all stages of the analysis, and, as new insights developed, theory was sought to help inform these.

**Results:**

We theorized that knowing in dental practice arises as actors translate embodied knowing through sayings and doings that anticipate but cannot predict responses, that knowing is constrained by the interactions of the practice but that the interactions at the same time are a collective bricolage—using the actors' respective embodied knowing to generate and solve problems together.

**Conclusion:**

Practices are ongoing ecological accomplishments to which people and things skilfully contribute through translation of their respective embodied knowing of multiple practices. Based on this, we argue that practices are more likely to improve if people and things embody practices of improvement.

## 1 INTRODUCTION

Efforts to improve outcomes from clinical encounters tend to take a narrow clinician or team‐centred approach and focus on propositional knowledge to the exclusion of other ways of knowing.^1^ Knowledge is a “thing” to be learned, retained, and applied as these actors solve problems in clinical encounters. Much of the academic writing about clinical practice assumes a “decision‐making” model—the doctor or dentist is seen as making diagnoses, selecting investigations/treatments, and perhaps eliciting patient's preferences—but underpinning all that is an assumed decision tree into which evidence can be fed (eg, 2, 3, 4, 5, 6). There are alternative ways to exploring what goes on as different actors come together that de‐centre knowledge from human actors, instead situating knowledge, or “knowing,” in the interactions between all actors in practice.^7^, ^8^

In these approaches, the knowledge is not an object retained within the boundary of human beings.^9^ Rather, knowledge is performative, generated in the moment, situated, interactive, and embedded within unique, ephemeral practices as actors perform.^10^, ^11^, ^12^, ^13^ Actors do embody representations of practice^14^ that we might called embodied knowledge, but knowing is *in* the practice. The actions of one actor influence (in an ongoing and reciprocal way) those of one or more other actors. Knowing in practice is ecological, one‐off episodes of interaction that involve people, things, and the environment at large.^15^, ^16^ Knowing is not the possession or responsibility of any one individual but is an ongoing, collective accomplishment.^10^

We are unaware of any studies that have explored how people and things generate knowing in practice in general dental practice. Thus, the purpose of this study was to explore how practice arises in the moment‐to‐moment interactions between general dental practitioners (GDPs), patients, nurses, and things within clinical encounters.

## 2 METHODOLOGY

We adopted a performative orientation where performativity is the constitution “of actors, meanings and roles through socio‐material practices.”^17^

D.H. is an academic GDP. His clinical experience includes working in general, community, and hospital dental services. The work described here is part of D.H.'s doctorate in evidence‐based health care at the University of Oxford. T.G., a general practitioner by training, has academic training in social sciences as well as in clinical medicine; she specializes in qualitative research in naturalistic (real‐world) settings.

### 2.1 Setting and sampling frame

We recruited two large general dental practices, providing predominantly National Health Service (NHS) treatment, as research sites. One was in a suburb of London and the other on the outskirts of a large town near London. The practices were selected because the owners were known to D.H. Attempts were made to increase the diversity of the sample by recruiting sites through social media and dental forums, but they were unsuccessful. Nevertheless, the resulting convenience sample exhibited a range of features, including years of experience of dentists, patient appointment types, and nursing experience.

All appointment types (eg, check‐up, emergency, treatment) were eligible for inclusion.

### 2.2 Recruitment

National Health Service ethical approval for this study was provided by the West of Scotland Research Ethics Committee, REC reference 17/WS/0064.

In the first instance, dentists and nurses in the two practices were invited to participate. After gaining written consent, a session for video‐recording was arranged. All adult patients with appointments to attend these were invited to participate by letter posted 2 weeks before the appointment with a covering letter from the dental practice and a participant information sheet. Patients were invited to contact D.H. by telephone or email before their appointment to discuss the study and to consent in principle to participate. Written consent was obtained on the day.

### 2.3 Data collection methods

**Figure 1:**
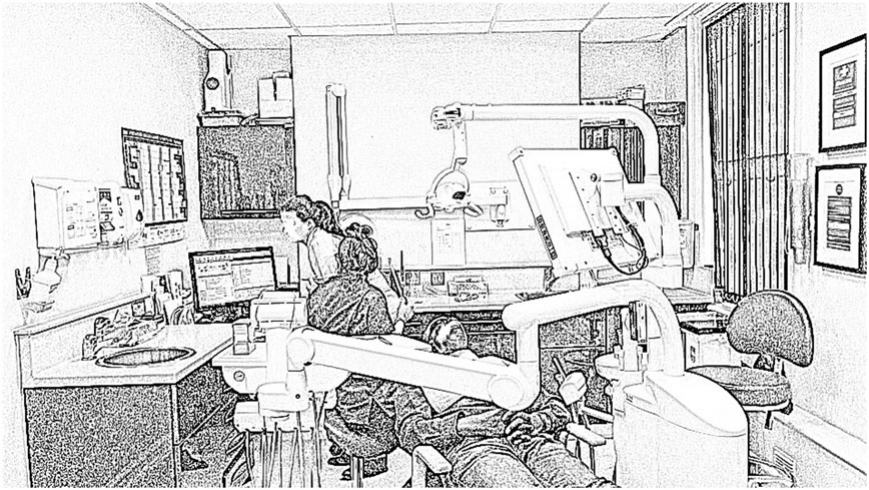
Typical field of view during video recording

We were interested in how practices unfold in real time, for which video is the optimal method of data collection.^18^, ^19^ The video camera was less obtrusive in small clinical spaces than a person, and the captured video could be watched and listened to repeatedly during analysis, rather than relying on notes captured in the moment. Over 4 days in August and September 2017, participants were filmed using a fixed digital video camera (Sony Handycam, HDR‐CX405) mounted on a tripod. It was placed in a position that was unobtrusive to patient and dental team, but which allowed patient, dentist, nurse, and computer to be captured in the field of view (Figure 1). D.H. set up the camera before the patient entered the room, pressed record, and then left. Once the patient had left the room, D.H. re‐entered it and stopped the recording. Video recordings were transferred to a password protected computer from the video recorder at the earliest possible moment, the original recordings deleted, and the SD card formatted.

In addition, D.H. informally observed the goings on in the respective dental practices and spoke with staff and patients during the periods of recording. Whilst not a formal part of the data analysis, anonymous notes were made of them to help situate the encounters recorded in the videos.

### 2.4 Units of study

Clinical encounters are often multi‐layered and complex phenomena, and so we chose our unit of analysis to be one or more *conjunctures* within each clinical encounter. Conjunctures are “a critical combination of events or circumstances” and may involve a brief or prolonged episode of interaction between actors.^20^ It is through studying very mundane and commonplace aspects of general dental practice within conjunctures that we intended to tease out an understanding of how knowing arose in practice.

### 2.5 Data processing and analysis

Informed by texts on the use of video in qualitative research,^19^, ^21^, ^22^ several analytic methods were used. These were used pragmatically and in combination. Detailed reflective notes were recorded at all stages of the analysis using electronic note‐keeping software (Evernote, Evernote Corporation), and as new insights developed, theory was sought to help inform these.

#### 2.5.1 Coding of videos

**Figure 2:**
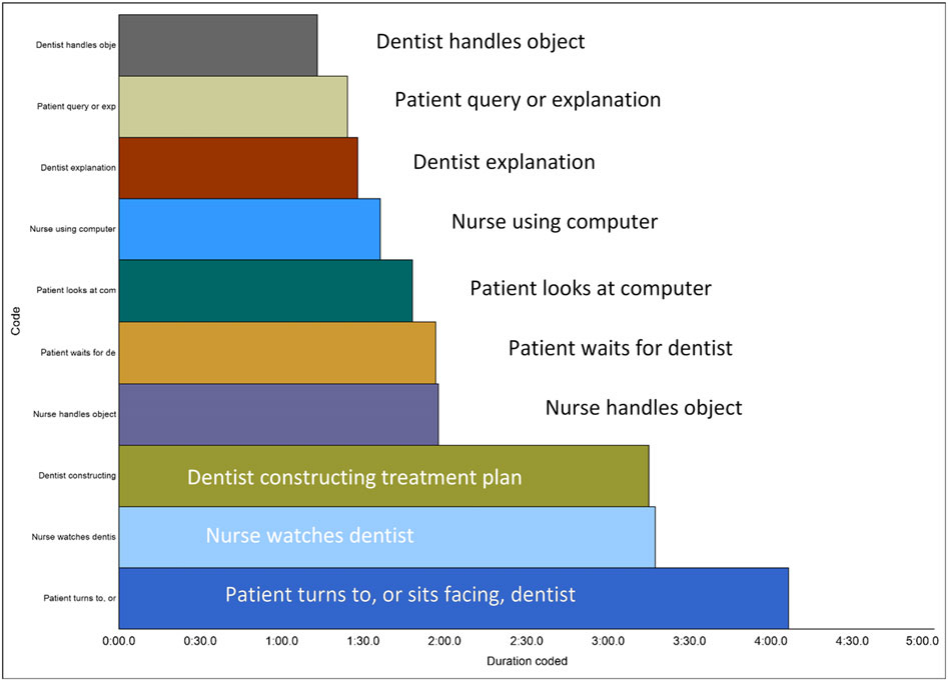
An example of coding activities from one clinical encounter. Duration is in minutes, seconds, and tenths of a second

This broad approach involved exploring what sorts of interactions occurred in the clinical encounters and to develop a sense of how much each actor was involved. All videos were imported into qualitative data analysis software (NVivo Version 11, QSR International Pty Ltd). Each was watched and re‐watched at least four times as D.H. coded the actions of each of the three human (patient, dentist, and nurse) and non‐human actors (eg, Electronic Patient Record, EPR). The coding distribution for the 10 codes with longest duration from one clinical encounter is shown as an example in Figure 2.

#### 2.5.2 Pencil drawings and transcript

We were inspired by Mavers' working paper on transcribing video^23^ to create a pictorial and narrative transcription of how humans and material objects interacted in the conjunctures. Goodwin created a drawing from a video still to show three girls at play,^24^ and Mavers used thick narrative to describe how children interacted with objects as they learned.^25^

**Figure 3:**
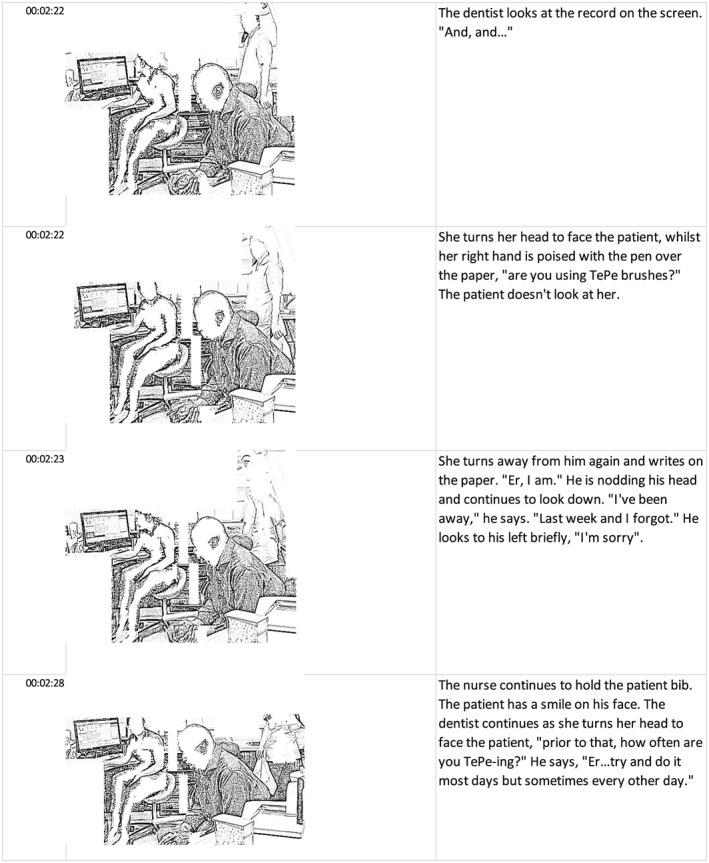
Excerpt from transcript of one conjuncture showing time from beginning of video when still was taken, the stills and associated narrative

Developing these approaches, D.H. took stills from one dentist's conjuncture whenever there was a change in physical orientation, eg, a dentist turning to a patient. The stills were turned into a pencil image using an Android mobile phone app (Photo Sketch Maker, Aero Tools) and, to bring focus to the interactions, all aspects of the image that were not involved directly were removed. The images were inserted into vertical consecutive cells in a spreadsheet (Microsoft Excel 2016). A narrative description of what was said and done for that section was inserted in the horizontally adjacent cell. A section from the transcript is shown below (Figure 3). The meticulous and detailed analysis of video data using pencil drawings allowed us to surface and explore in‐the‐moment interactions as a series of “freeze frames,” thereby allowing us to develop a highly innovative analysis of knowing in practice.

#### 2.5.3 Dynamic transcription

**Figure 4:**
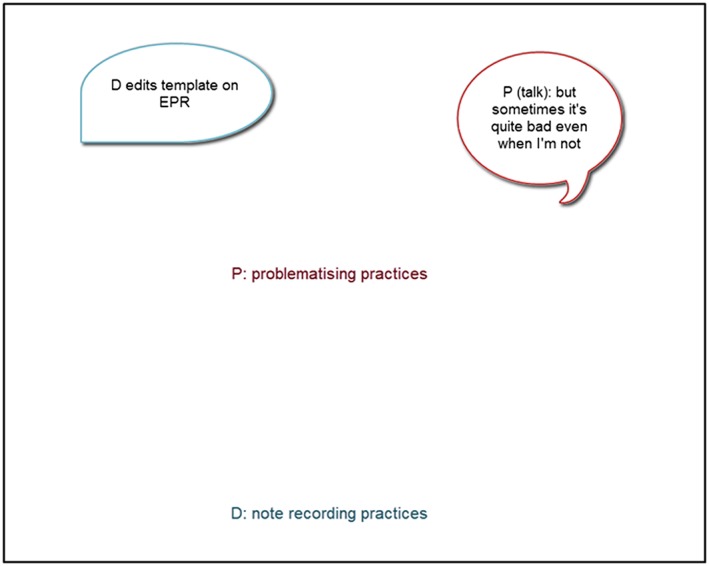
Still 1 from dynamic coding of a conjuncture. Text in bubbles are enactments. Floating text is knowing of practices embodied and translated as inferred from the saying and doing by D.H.

**Figure 5:**
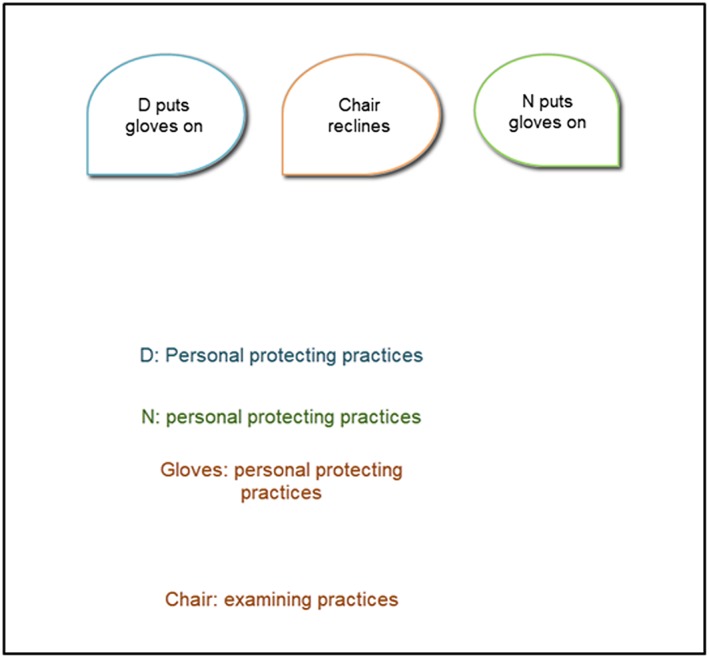
Still 2 from dynamic coding of a conjuncture. Text in bubbles are enactments. Floating text is knowing of practices embodied and translated as inferred from the saying and doing by D.H.

We were keen to explore the dynamism of the knowing being enacted. Using a video editing software (Camtasia Studio 8, Techsmith Corporation), D.H. transcribed what was said, the physical actions and the embodied knowing he inferred from the actions. Two stills from the video are shown as examples in Figures 4 and 5. The time each element was on the canvas was a minimum of 1 second or for as long as the action occurred, if longer.

## 3 RESULTS

### 3.1 Description of data sets

The data set included the raw footage of 22 appointments that totalled eight and a half hours, a single clinical encounter transcribed in full, with stills converted into pencil and another whole encounter transcribed into the video of dynamic coding. D.H. recorded the equivalent of approximately 160 A4 pages using digital note keeping software (Evernote, Evernote Corporation).

Clinical encounters took the following general form: GDPs would check a patient's dental record on a computer in the surgery either prior to asking the nurse to bring the patient into the room or as the nurse was doing so; the patient entered the surgery, there was an exchange of pleasantries, and the patient sat on the dental chair; the GDP and patient then established or confirmed the reason for the visit (eg, to address a problem or to place a filling) followed by a brief or longer discussion about this; the patient would then recline in the dental chair and a degree of interaction between GDP, patient, nurse, and instruments would centre around examination or intervention within the mouth, including the taking of radiographs; the patient would be sat up in the chair, and there would be a brief or long discussion relating to the prior phase including diagnoses and possible interventions; the patient would leave, and the GDP would write notes into the patient's record as the nurse made the surgery ready for the next patient.

What we focus on below is not the embodied learning GDPs develop per se, which is often the focus of, for example, anthropological studies of apprenticeship,^26^ but on the moment‐by‐moment interactions to which GDPs, other people and things translate their embodied or embedded knowing.

#### 3.1.1 Dialogism in actions

In these encounters, relationships between people and things were established as temporary phenomena through discussion and through prodding and probing, reading and writing, seeing and hearing. That is, as one actor said or did something, the other observed, listened, or felt and, in due course, acted or spoke themselves. This might involve listening to a question and responding, or a patient seeing an instrument orientating towards their mouth and opening it.

Bakhtin wrote of the *dialogic relationship* that was essential to what he called utterances. Utterances are only utterances because there is an anticipation of a responsive understanding by another.^27^ In these clinical encounters, in addition to dialogism in utterances was dialogism in actions. Actors said and did things in anticipation of a responsive understanding by another.

As a GDP picked up a dental mirror and probe to check gum health, for example, the nurse would move to the computer and ready herself to record the results without the GDP saying anything. The GDP would then slide their chair and position themselves behind the supine patient's head. The GDP would raise the probe and dental mirror over the patients face, and the patient almost always opened their mouth with no verbal prompting. If they did not, then the GDP might place a finger on the lower jaw or, rarely, ask the patient to open their mouth. The GDP, nurse, and patient all appeared to be anticipating the other's—and the things' (computer, probe, chairs)—actions and reactions. This is not to say that all the reactions were correctly anticipated. For example, a GDP had asked a patient to lift their chin, and the patient lifted his whole head forward, essentially tilting his chin into his chest, the opposite of what the GDP had anticipated. Nonetheless, the GDP had anticipated an action, the patient had acted and in turn anticipated a reaction from the GDP (that they would continue with the examination). The GDP, though, reacted with another action to align the patient's head as they needed it before they continued as the patient had anticipated.

The anticipation of others' actions suggested to us that the actors had embodied understandings of how people and things had reacted in their experience of historical practices. That is, they expected that something would happen if they did something because they had an embodied knowing of previous interactions in which something had, even if it was not in this or any other clinical space.

Given the speed with which they did so, this was mostly done unconsciously and working skilfully to translate their embodied knowing into this practice. They need not have had an experience that followed a similar sequence of interactions between people and things. They may have, instead, translated something they had parsed from those previous experiences. Marchand has suggested that apprentices parse components of actions they observe their teachers do into “motor representations” of how to do things themselves, which they attempt to enact.^14^ It seems that experience of any other practices might be parsed into embodied knowing, even when learning how to act as a patient. This is to say that there will be elements of practices previously experienced (in this space, in another clinical space or somewhere non‐clinical) that can be translated in some way into the ongoing practice. For example, no matter which instruments were raised over a patient's face by the GDP, the patient almost always opened their mouth. From some experience in the past, the patient had come to embody an understanding of how to respond to the appearance of things (including the GDP's hands) over their face.

Anticipation of others' actions is common to many aspects of life. In relation to people, we have called this dialogism, but in relation to objects, the anticipation is of how the object might be used, what Heidegger would call being “ready at hand.”^28^ A GDP, for example, picked up a dental mirror, which is normally used to mirror teeth and other tissues that the GDP cannot see directly. On this occasion, though, he anticipated its potential to elicit pain from around a tooth by turning it through 180° and tapping the tooth with a sharp knock with the handle. The patient responded with an “ow!” and the task of the conjuncture came to focus on working out why.

The anticipation of a response and the response (with its anticipated response, and so on), whether mediated through the spoken word, through touch, sight, sound or smell, through objects or not, gave rise to an interaction in which shared knowing appeared to exist for the moment. In the interaction between them, the GDP, patient, and dental mirror handle, generated an act of knowing. If the GDP had not anticipated there might be a response from tapping the tooth, he may never have applied the tap to the tooth. If he had not anticipated the patient responding (eg, if she were under general anaesthesia) the knowing shared in that moment that something was “wrong” with that tooth would not have arisen. If the patient anticipated that something terrible would happen if she said “ow” and so did not, then the act of knowing would have been different. Conjunctures, in which knowing arose, went on through multiple actions that anticipated—but could not predict—reactions.

The embodied knowing we inferred could be analytically (and only analytically) separated into that which is closely aligned with the ongoing conjuncture, similar to what Greenhalgh and Stones called conjuncturally specific knowledge,^29^ and that which is less closely aligned with the conjuncture, but which nonetheless translates into it, which they called general dispositions and Bourdieu called *habitus*.^34^ Whether the embodied knowing was conjuncturally specific or not, the translation almost always was. Each person or thing, in doing or saying something that interacted with others within the conjuncture, translated whatever embodied knowing they had at that time in a way that meant the conjuncture went on.

**Figure 6:**
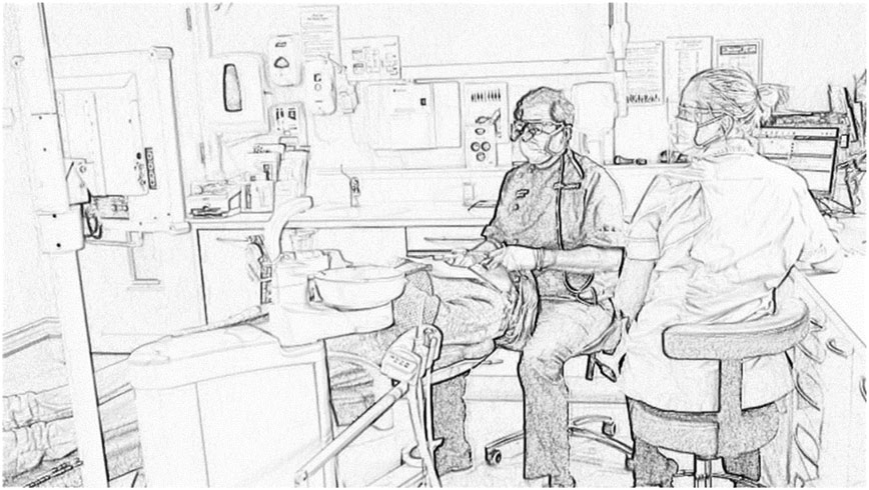
Still showing a dentist about to take a photo of a patient's upper central incisor

We illustrate this with Figure 6. It shows a dentist about to take a photo of a patient's upper central incisor, which had chipped. Not a word was said by the dentist about taking the photo. He had just finished looking at the tooth, edged the chair back a fraction, and then reached for the camera. As he brought it over the patient's mouth, she bared her upper teeth by raising her lip, the nurse moved her right hand to the computer mouse and, as the dentist did, turned her head to the monitor above the patient. At this moment, each of the people translated their embodied knowing in actions that meant their respective knowing aligned, the photo could be taken, and the conjuncture could go on.

### 3.2 Constraint and power

The practice and, therefore, the knowing arising in it, was suffused with power or influence^30^, ^31^ as the different actors attempted to translate their respective embodied knowing (including of influencing others) into the ongoing practice.

People and things did what they could do within the interactions, which were bound by space and time. However, the relationships established through interaction moment‐by‐moment constrained the possible translations of knowing that could occur. For example, at one moment in the conjuncture about dental pain mentioned earlier, the dentist was facing the computer as the patient described her pain. She had not mentioned where it was but pointed to the upper right side of her face. Because the dentist was not looking at her, it meant that the knowing her action attempted to translate failed. The translation of the patient's embodied knowing was constrained by the in‐the‐moment relationship between her, the GDP, and the computer. Similarly, in an encounter involving another GDP and patient, the GDP had set the chair in motion to go supine. The patient had been complaining that a previous filling was too high. He lifted his head from the dental chair rather than lying supine as all other patients did, as he continued protesting. The patient's translation of his discomfort and dissatisfaction was constrained by his interaction with the chair and the GDP. The GDP's translation of the planned activity (restoration of another tooth) was constrained by his interaction with the patient and the filling in his mouth. A final example was the bodily positioning of a patient who looked at the wall rather than the GDP as she explored his oral hygiene habits. Her confidence in addressing this appeared to wane. This was in stark contrast to the next patient who turned in the chair and attended to her with evident interest as she explored his oral hygiene habits.

Most of the time, the constraints did not manifest so obviously, but all appointments were constrained by interactions with the other people, the equipment, the space, and time. Actors were constrained by their environment. This, we consider to be part of the situatedness of action. Whatever the representations the different actors embodied about these practices, what we could call ostensive practices, were quite different to the situated performed practice because of these constraints and the reactions of others.

Translation of knowing of practices inscribed in, for example, the computer software could also be constrained by the conjuncture's interactions. Thus, a template of questions from NHS England that was embedded within the check‐up page on the computer software prompted some dentists to ask several questions about patients' habits related to risk of oral diseases, eg, their sugar, tobacco and alcohol consumption, and oral hygiene regime but did not prompt others. The dentists who read the list out tended to be more focused on the computer than the dentists who did not but who attended more to the patient.

The template from NHS England is an example of an expert system, which is a “disembedding mechanisms because … they remove social relations from the immediacies of context.”^32^ Through it, members of that and other organizations attempted to constrain the activities of GDPs. Sometimes, the interactions allowed this other actor to induce different actions (one in which a dentist asked questions about behaviour) whilst others did not. Through their direct (discursive practices and touch) or indirect actions (via intermediaries such as artefacts), each actor attempted to influence the ongoing interactions, induce different knowing to co‐exist, and therefore shape the unfolding practice.

### 3.3 Collective bricolage

Practice was accomplished through the adaptive translation of knowing from the different people and the things they interacted with. People and things did what they could, given the interactions between them, and generated actions (eg, creating a problem, creating a solution) for the conjuncture to go on, to come to an end (at least for now) and for the constellation of actors to disperse. Like the bricoleur who, with their limited skill set and tools, solves problems as best they can,^33^ together people and things generated and solved problems in a collective bricolage. Patients used the words they knew from non‐dental experiences, for example, to describe their dental experiences. They offered focused reflections on experiences they had, such as taking medication when they shouldn't because of a medical diagnosis. They offered up proposals of what could be wrong, of what prior treatment they had received that might be relevant, of their habits and their symptoms. GDPs observed and listened, made suggestions of diagnoses and treatment approaches, used the instruments and materials at hand in different ways (the mirror handle mentioned previously, a probe designed for screening only to make diagnoses), and took radiographs. All of them, working together, using their respective capacities, created the practice in that moment.

An example is the way in which a problem was fashioned within a check‐up appointment to create a conjuncture related to relieving a denture that was pressing on a patient's palatal mucosa (the skin on the palate). The patient mentioned that there was a sore patch under her denture, which she believed related to the denture. The dentist listened and, as part of a check‐up, looked at the denture and the mucosa under it. After a few moments, he suggested that a tooth on the denture was pushing into the mucosa because he could see a red area that related to the position of the underside of the tooth. He explained he would adjust the denture, which the patient acceded to, picked up a handpiece with a bur designed to trim the acrylic from a denture and removed the offending part as the patient watched. When replaced, she said it felt fine. Each of the actions was attempt to translate embodied knowing as best they could so that they could generate the problem and solution collaboratively. The skill was in the respective and ongoing translations of knowing within the interaction by each actor. GDPs did not act alone in their surgeries. They enacted practices with the patient, nurse, and things.

## 4 DISCUSSION

This study of eight GDPs interacting with patients has shown that knowing in general dental practice occurs as transitory interactions between humans and things within clinical conjunctures. The relationships between people and things within these interactions allow for a temporary shared knowing to co‐exist. Each relationship was soon replaced with another as participants acted and reacted over time and space. The possible translation of embodied or embedded knowing through actions was constrained by the ongoing interaction. Thus, the knowing that could arise was dependent not only on the embodied knowing but on the translation of this in such a way that it could contribute to the collective knowing in practice. Finally, the various participants worked together to solve the problems and create solutions as best they could in a collective bricolage.

In this study, we have sought to build on previous work that suggested health care workers require many more sources of knowledge than that provided by research 34 and are guided by internal, tacit and socially constituted guidelines, or *mindlines*, and that “knowledge in practice” is the use of this complex knowledge in the clinical context.^35^, ^36^, ^37^ The concept of knowledge in practice differs from the social anthropological, organizational, and educational literature's “knowing in practice.”^11^, ^12^, ^13^ The former seems more concerned with some‐*thing*, a practical knowledge used by clinicians *as* they practice. The latter is more concerned with some‐*doing* and stresses the socio‐material, ephemeral, provisional, and collective nature of knowing *in the practice*. As Marchand writes, “… acts of making knowledge are always and necessarily realised *in* interaction with others and with the world.”^38^ Practice, and the knowing that arises in it, is ecological. This paper contributes to the literature of clinical practice by suggesting that for conjunctures to go on (ie, for clinical problems to be generated and, hopefully, solved), multiple temporary relationships between people and things need to be established in such a way that knowing relevant to the conjuncture can align. This shifts the focus from concentrating on what a clinician “knows” to the knowing they play a part in generating, which is situated in their environment and with the people and things there. Harris, writing from an anthropological perspective, implores us to remember that knowing is bound up with the world and that, “a person does not leave their environment to know, even when she is dealing with the most abstract of propositions.”^13^ GDPs, patients, nurses, and things, we suggest, only know in the moment‐by‐moment unfolding of clinical encounters. They may leave with a representation of this encounter as they may an encounter elsewhere, with an interpretation they come to embody, but they only know this practice as they interact with each other.

Relevant here, also, is the concept of *Habitus*. Bourdieu wrote that it is “… embodied history, internalized as a second nature and so forgotten as history ‐ is the active presence of the whole past of which it is the product.” Actors acted based on their embodied or embedded knowing of multiple practices across time (or history) and space. We do not think of *Habitus* as a product but a dynamic embodied phenomenon that generates “practical hypotheses based on past experience.”^39^

We have intentionally offered a description of practice that makes it complex, even when addressing routine and mundane clinical decisions and interactions. Evidence‐based health care intended to bring research, clinical expertise, and patient values and aspirations together but has tended to focus on getting practitioners to change their actions, or patients to change their behaviours, to be more aligned with what researchers suggests will improve patients' wellbeing, the efficiency of care or some other outcome of interest. We think, based on our findings and the epistemology of practice to which we contribute, that there is a need to pay much more attention to the complexity of practices and to recognize that what people translate into ongoing practices is not propositional knowledge but embodied knowing of practices they have either experienced personally, observed or, as other work we have yet to publish suggests, through vicarious experiences, i.e., others' stories of their practices.

The role of the GDP in practice is as one of several who together shape the conjuncture, in contrast to traditional decision‐making models in which the professional is often the only one conceived as bringing (a usually “scientific”) knowledge to an encounter so that they can diagnose and treat problems. Several authors have suggested ways of thinking about professional knowing that are alternative to this. Montgomery, in *How doctors think*, wrote that whilst scientific information can help reduce uncertainty, ultimately medicine is still a practice.^40^ Mol, in *The body multiple: Ontology in medical practice*, wrote that the doctor's knowing of a disease was but one of several. The patient, the pathologist, and others with a stake in living with, diagnosing, or managing a disease each had their own knowing of that disease.^41^ Billett argued that knowing in practice is the construction of knowing in a social world rather than knowledge as a cognitive thing. Practice in a given situation requires knowing that arises in the situation that can influence the way in which skills are enacted.^11^ We want to emphasize that whilst the dentist brings an embodied knowing to the encounter, so too does the patient, other people, and material things. Professional knowing is more than that which is embodied by “the professional.” It is instead a dynamic, reflexive, and ongoing activity to which the patient, other people, and many things, each translating their respective knowing, contribute. Professional knowing cannot exist out of the performance of professional practice or without the patients and other actors that are central to that practice.

If we are concerned with the quality of clinical care we need to think beyond propositional knowledge, which takes the form of research publications, systematic reviews, and guidance. The care that arises (or not) in practice is one that arises from an embodied knowledge or knowing, and which is translated into interactions that constrain it on the one hand and generate other knowing on the other. Whilst communication is an important component of the interaction between people and things, there are other interactions that interfere with or promote the translation of actors' knowing. Practice is ecological—it takes place in an environment where people and things interact through restricting, touching, pushing, prodding, smelling, marking (instruments), images, and sounds. And people learn to act within practice much as apprentices do, “through observation, mimesis and repeated exercise.”^42^ Clinical care arises through the non‐propositional, through the translation of knowledge embodied or embedded in bodies and things. The embodiment of learning from multiple practices, each in their own environment and with all the parsed learning they take from that, is what GDPs, patients and nurses draw on to act and react in ways that are relevant to the ongoing conjuncture. If the practice of clinicians and teams is to be evaluated, this needs to be done in relation to their respective ecology. A clinician's activities cannot be evaluated out of relation to their environment. If we want to help improve practice, rather than focusing on disseminating propositional knowledge in the form of guidelines, we should focus on finding ways to allow dental teams, patients, and things to take part in and contribute to practices *as they improve*. Or at the very least, offer vicarious experiences in the form of narratives of how others practicing in similar ecologies improved their practices.^43^ They may then come to embody knowing of practices not as accomplished “good practice” but as the active phenomena they are. As others have shown with apprenticeship, we learn to embody knowing of practices by observing, having a go, and correcting what we do. We suggest that practices are more likely to improve if we embody knowing of practices of improving.

### 4.1 Limitations

D.H. was the only person to analyse the data in depth. Sections of video were analysed with T.G. and interpretations of what was happening discussed regularly throughout the analysis. The concept of practices taking place within a nexus of other practices and the translation of an embodied knowing into an ongoing practice means that the practice of interacting with the data involved translating into it an embodied knowing of other practices. D.H. (as a dentist) had an embodied knowing of some dental practices that were very similar to those being observed, whereas T.G. (as a non‐dentist) embodied a lifetime of GP practices and of theoretical writings relevant to practice. Thus, the detailed analysis and interpretation of the data were appropriate for D.H. to do alone, with T.G. bringing a critical perspective to the findings based on her theoretical and GP experience.

### 4.2 Conclusion

Practices are ongoing ecological accomplishments to which people and things skilfully contribute through translation of their respective embodied knowing of multiple practices. Based on this, we argue that practices are more likely to change if people and things embody practices of improvement.

## CONFLICT OF INTEREST

None declared.
